# Premorbid school performance trajectories in patients with treatment-resistant schizophrenia prescribed clozapine in the public health system in Chile: a case-control study, 2007–2020

**DOI:** 10.1017/S0033291725101062

**Published:** 2025-07-21

**Authors:** Jose Conejeros-Pavez, Javiera Vasquez, Camila Diaz, Cristian Mena, Juan Undurraga, Alfonso Gonzalez-Valderrama, Susana Claro, Eduardo A. Undurraga, Nicolas A. Crossley

**Affiliations:** 1 School of Government, Pontificia Universidad Católica de Chile, Santiago, Chile; 2 Department of Psychiatry, School of Medicine, Pontificia Universidad Católica de Chile, Santiago, Chile; 3 Pharmacovigilance Program, Dr. José Horwitz Barak Psychiatric Institute, Santiago, Chile; 4 Early Intervention Program, Dr. José Horwitz Barak Psychiatric Institute, Santiago, Chile; 5 School of Medicine, Finis Terrae University, Santiago, Chile; 6 Department of Neurology and Psychiatry, Faculty of Medicine, Clínica Alemana Universidad del Desarrollo, Santiago, Chile; 7 Research Center for Integrated Disaster Risk Management (CIGIDEN), Santiago, Chile; 8 Department of Psychiatry, Universidad de Antioquia, Medellín, Antioquia, Colombia; 9 Department of Psychiatry, University of Oxford, Oxford, UK

**Keywords:** global health, long-term outcomes, premorbid academic achievement, psychiatry, treatment-resistant schizophrenia

## Abstract

**Background:**

The premorbid phase of treatment-resistant schizophrenia (TRS) may reveal underlying mechanisms and inform early interventions. According to the neurodevelopmental hypothesis, treatment resistance may be linked to pronounced developmental impairments. We examined school grades and attendance trajectories in children who later developed TRS.

**Methods:**

This case-control study analyzed school grade point average and attendance among all individuals born after 1990 and started on clozapine in Chile’s public health system as a proxy for TRS. Control groups included children later diagnosed with treatment-responsive schizophrenia, bipolar disorder, and unaffected classmates. Linear mixed models accounted for individual and school-level confounders.

**Results:**

We included 1072 children (9929 observations, 29.3% female) subsequently diagnosed with TRS, 323 (2802 observations, 25.7% female) with schizophrenia, 175 (1784 observations, 53.8% female) bipolar disorder, and 273,260 (533,335 observations, 47% female) unaffected classmates. Children who later developed TRS had worse grades across levels than their classmates (−0.26 SD [−0.2, −0.4]), but not treatment-responsive schizophrenia. All severe mental illness groups showed grade declines in later school levels, with TRS showing steeper linear decline than treatment-responsive schizophrenia (*group*×*age* of −0.03; 95%CI −0.04, −0.01) and steeper quadratic decline than bipolar disorder (*group×age* ^2^ of −0.005; −0.01, −0.001). Attendance declined over time in the two groups developing schizophrenia compared to their classmates. Those developing TRS experienced the sharpest drop (*group×age* compared to schizophrenia −0.03; −0.05, −0.01 and bipolar disorder −0.027; −0.049, −0.006).

**Conclusions:**

TRS may stem from a more aggressive pathological process or pronounced late-maturation abnormality, rather than an early premorbid impairment, suggesting an intervention target.

## Introduction

Schizophrenia can have a varied impact on people, ranging from those who recover successfully, to those who struggle to regain their functioning (Jääskeläinen et al., 2013). Individuals with treatment-resistant schizophrenia are a group that is specifically at risk of poor functional outcomes (Iasevoli et al., 2016). Treatment resistance can present in up to 23% in the first five years of illness (Siskind et al., 2022). Research into the premorbid characteristics of individuals who develop treatment-resistant schizophrenia can provide insights into its pathological mechanisms and allow for their early identification (Potkin et al., 2020).

The neurodevelopmental theory of schizophrenia (Murray & Lewis, 1988) suggests that treatment-resistant schizophrenia is driven by a more severe neurodevelopmental alteration, resulting in the poorest pre-morbid functioning among individuals with schizophrenia. Previous studies have shown worse retrospective functioning (Chan et al., 2021; Üçok et al., 2016; van Hooijdonk et al., 2023) and lower IQ scores based on National Adult Reading Test (Legge et al., 2020), although these findings are limited by their retrospective nature.

Educational attainment, defined as the completion of various levels of education and considered less susceptible to biases in retrospective recall, has also been examined. Clinical studies have described a significant association between fewer years in education and future treatment resistance (Smart et al., 2022; van Hooijdonk et al., 2023), though not all do (Legge et al., 2020; Sharma et al., 2021) Population-based cohorts using national registers report mixed results as well: a Swedish study showed significantly lower educational attainment in those with treatment-resistant schizophrenia compared to non-resistant cases (Kowalec et al., 2021). At the same time, a Danish study found that individuals with treatment-resistant schizophrenia often completed education levels higher than primary school (Wimberley et al., 2016). However, educational attainment can be affected by the disruptive effect of the illness itself (Crossley et al., 2022), leading to absenteeism and school dropout (John et al., 2022). As such, reduced educational attainment may partly reflect the illness’s early onset and disabling effects in youth, as is usually the case with treatment resistance rather than neurodevelopmental deficit.

Performance tests and academic grades at various developmental stages may offer additional insights into premorbid cognitive development, though not independent of school attendance. Kowalec et al. (2021) examined grades at year nine in children who later developed psychosis and found that grades were slightly lower in those who developed resistance. However, this result was not robust to changes in the definitions of treatment resistance from the register. They also reported a decrease in IQ at age 18 in males, a finding not replicated in a smaller study in Israel of similarly aged men and women undergoing military drafting (Caspi et al., 2007). These variations in premorbid cognitive functioning across developmental stages highlight the need to study the progression of premorbid cognitive deficits (Reichenberg et al., 2010).

To understand treatment resistance antecedents, we retrospectively examined school performance and attendance in 1072 individuals in Chile who began clozapine treatment within the public health system. This group included all born after January 1990 and prescribed clozapine by January 2020, as recorded in Chile’s national pharmacovigilance program for clozapine (Mena, Nachar, Crossley, & González-Valderrama, 2019), which mandates registration for public health service patients. We compared their academic achievement with three control groups: classmates without severe mental illness, individuals who later were diagnosed with schizophrenia who were treatment-responsive, and individuals who developed bipolar disorder. This latter group represents individuals who developed the disruptive effect of severe mental illness without presenting the same range of premorbid cognitive difficulties (Mollon & Reichenberg, 2018). Unlike prior studies, we analyzed children’s grades and school attendance rates from ages 7 to 18, capturing a range of academic trajectories that could inform the underlying mechanisms of premorbid impairments. We hypothesized that children later prescribed clozapine would show more pronounced early-age impairments compared to all other groups, with impairments not solely attributable to absenteeism.

## Methods

### Study population and design

We conducted a case-control study using data on educational performance and school attendance from individuals diagnosed with treatment-resistant schizophrenia in Chile between 2007 and 2020. We included all individuals born after 1990 who began clozapine treatment in Chile’s public health system before January 2020 and had at least one year of education outcomes (grade and/or attendance). Following other studies, we used clozapine prescription as a proxy marker of treatment resistance (Ajnakina et al., 2018). According to Chilean national guidelines, clozapine is prescribed exclusively for treatment resistance (MINSAL, 2017). Around 80% of the Chilean population receives healthcare in the public health sector (Aguilera et al., 2014). Individuals prescribed clozapine in these institutions are registered in the National Clozapine Hematological Monitoring System, from which cases were identified (Mena, Nachar, Crossley, & González-Valderrama, 2019).

We also included three control groups. The first consisted of individuals born after 1990 who were diagnosed with a first episode of schizophrenia between 2007 and 2020 at the Psychiatric Institute Dr. José Horwitz, the main mental health referral center in Chile, located in Northern Santiago, and who had not started clozapine by January 2020. The second group comprised individuals diagnosed with bipolar disorder at the same institution during the same period, who had not received clozapine. This group is known to have fewer premorbid cognitive deficits than those developing schizophrenia, but can still experience a similar disruptive effect on young people. This control group helped to differentiate changes in grades due to cognitive difficulties or illness disruption (e.g., non-attendance, non-engagement due to prodromal symptoms), and also provided the reader a means of validating our cohort´s results from the Global South against existing literature. As in previous research (Mena et al., 2018), diagnoses were obtained from the mandatory case notification register as per Chilean law (Letelier & Bedregal, 2006), and are based on the treating clinician’s assessment. The final control group included all the classmates of the individuals from the first three groups who attended the same school in the same year, serving as a healthy control group.

We integrated health data from the Ministry of Health with administrative data on student attendance and grades from the Ministry of Education, covering the period 2002–2020. The Chilean education system provides nearly universal coverage. Until 2002, compulsory education extended up to 8th grade (age 14). In 2003, it was expanded to include up to 12th grade (age 18), encompassing all participants in our study. To ensure secure data integration, we implemented safeguards preventing personnel handling identifiable educational data from accessing health-related information. Researchers worked only with anonymized data. Details are provided in the Figure S1.

### Variables

Our primary outcome variables were academic achievement, measured by grade point average, and school attendance.

We normalized all grades and attendance rates for each school and level, using *z*-scores to compare a child’s performance with that of peers at the same school. This normalization accounts for socioeconomic status, which is associated with high socioeconomic segregation in the Chilean educational system.

Academic levels are described from 1st grade to 12th grade, which generally correspond to children aged 7–18 years old. Sex was obtained from the government records. School vulnerability is defined by the Chilean Ministry of Education and reflects the proportion of children who are eligible for free meals based on their socioeconomic status.

### Analyses

We analyzed the data using mixed linear models independently for each main outcome (grades and attendance), adjusting for school effects, sex, age, and year retention as confounding factors, and accommodating repeated measures and a variable number of observations for each participant. We initially compared each of the three patient groups with their healthy classmates (reference) using the following model (Eq. 1):
(Eq. 1)

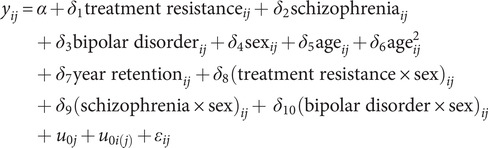

where 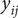 is the normalized score (z-score) for grades or attendance for student 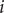 at school 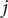 and 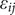 is an error term. The model includes confounder’s sex and age (linear and quadratic), along with a dummy variable for *year retention*, indicating whether the student had repeated a school year. This practice was common in Chile for failing students until 2019, and grades often improved upon the second attempt. The model also includes two random intercepts: 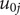 for school *j*, and 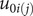 for individual *i* nested within school *j* that accounts for school-specific confounders as well as repeated measures. To examine the potential effect of the group on academic trajectories, we compared the fit of Eq. 1 (Model 1) with two sequential models examining changes in the trajectories of the outcomes (grades or attendances): Model 2, which included an interaction term between disorder and age; and Model 3, which added an interaction term between disorder and age^2^. This approach allowed us to test whether the trajectories of outcomes differed between individuals with disorders and their unaffected classmates. We also included a *group × sex* interaction term to examine differences in the trajectories according to sex.

After examining all disorders compared to the unaffected classmates, we then built two similar models comparing treatment-resistant schizophrenia with treatment-responsive schizophrenia and bipolar disorder.

Sensitivity analyses restricting our national sample of children who later developed TRS to those who attended school in the capital city of Santiago, to match them geographically with the other two groups of children who developed severe mental illness, are reported in the Supplementary Information.

We also performed mediation analyses as described in the Supplementary Information Methods to explore the potential role of changes in attendance on grades.

All analyses were conducted using R version 4.4 with the lme4 library (version 1.1.35.5) to estimate mixed models with maximum likelihood estimation.

This study was approved by the Ethical Scientific Committee of the Servicio de Salud Metropolitano Norte (AE-010/2021).

### Role of the funding source

The funder of the study had no role in the study design, data collection, data analysis, data interpretation, or writing of the report.

## Results

### Study population

Our sample included 1072 children (9929 observations) subsequently diagnosed with schizophrenia and prescribed clozapine. This corresponded to 31.3 prescriptions per 100,000 persons (people aged 15–29 in Chile in the public health system), a comparable rate to that observed in other countries (Bachmann et al., 2017). Additionally, we included 323 children (2802 observations) later diagnosed with schizophrenia and 175 children (1784 observations) with bipolar disorder, none of whom were prescribed clozapine. The sample also included 273,260 children who were classmates of the children in the previous groups at the time of each observation (median number of observations per case observation of 36, a total of 533,335 observations) and who had not been diagnosed with schizophrenia or bipolar disorder. Table 1 shows the characteristics of the sample.

**Table 1.** Characteristics of the participants included

Variables	Treatment-resistant Schizophrenia	Treatment-responsive Schizophrenia	Bipolar disorder	Controls (classmates)
Number of children	1072	323	175	273 260
Number of observations	9929	2802	1784	533 335
Percentage of girls	29.3^a^ ^,^^b^	25.7^a^	53.8^a^	47.0
Number of observations according to school level (*n*)				
1st grade (age 7)	647	126	93	28 769
2nd grade (age 8)	742	163	111	33 661
3rd grade (age 9)	872	208	133	40 708
4th grade (age 10)	953	233	149	45 366
5th grade (age 11)	966	238	162	48 029
6th grade (age 12)	1041	293	175	52 242
7th grade (age 13)	1005	307	175	53 830
8th grade (age 14)	911	287	165	48 828
9th grade (age 15)	1075	346	185	69 784
10th grade (age 16)	655	227	154	44 281
11th grade (age 17)	728	247	167	47 906
12th grade (age 18)	334	127	115	19 911
Vulnerability index for schools [z-scores, 95% CI]	0.44 [0.39–0.49]^a^ ^,^^b^	0.52 [0.43–0.62]	0.81 [0.69–0.94]^a^	0.54 [0.537–0.543]
Nr. of different schools attended per child [median, interquartile range]	3 [2–4]^a^	3 [2–4]^a^	3 [2–4]^a^	1 [1–1]
School year retention (% observations)	9.49^a^	8.64^a^	4.65	4.00
Age of participants at last follow-up [median, interquartile range].	25 [22–27]	27 [23.5–30]	25 [22.5–27]	24 [21–27]

*Note*: Vulnerability indices for schools are represented as z-scores, with higher values indicating lower vulnerability.

a Statistically significantly different from unaffected classmates in post-hoc testing (*P* <0.01).

b Statistically significant difference between treatment resistant schizophrenia and treatment responsive schizophrenia in post-hoc testing (*P* <0.01).

The proportion of observations from girls was statistically different across groups (χ^2^ = 474.28, degrees of freedom (df) = 3, *P* <0.0001), with the lowest proportion in the group later developing treatment-responsive schizophrenia, then those with treatment-resistant schizophrenia, healthy classmates, and the group developing bipolar being the one with the highest proportion (all post-hoc comparisons *P* <0.001 using Holm-correction). Groups attended schools with different levels of vulnerability (ANOVA *F* = 97.99, df = 3, *P* <0.0001). The highest levels of vulnerability were in schools attended by those developing treatment resistance, and the lowest in those developing bipolar disorder (all pairwise post-hoc comparisons between the two latter groups using Tukey *P* <0.001). Children who later developed severe mental illness were more likely to attend a higher number of different schools compared to their unaffected classmates (Kruskal-Wallis Η= 6720.8, df = 3, *P* <0.0001; with post-hoc Holm test *P*<0.0001 compared to unaffected classmates). The number of observations from children who had repeated the school year also differed between the groups (χ^2^ = 888.59, degrees of freedom (df) = 3, *P* <0.0001), being higher in those developing schizophrenia (similarly for treatment-resistant and treatment-responsive) compared to those presenting bipolar disorder and the unaffected classmates. Table 1 also provides the participants’ age at the end of follow-up in January 2020 for each group to provide an idea of the population studied, as well as the period at risk for those not diagnosed with severe mental illness or not started clozapine.

### Academic achievement

**Figure 1.**
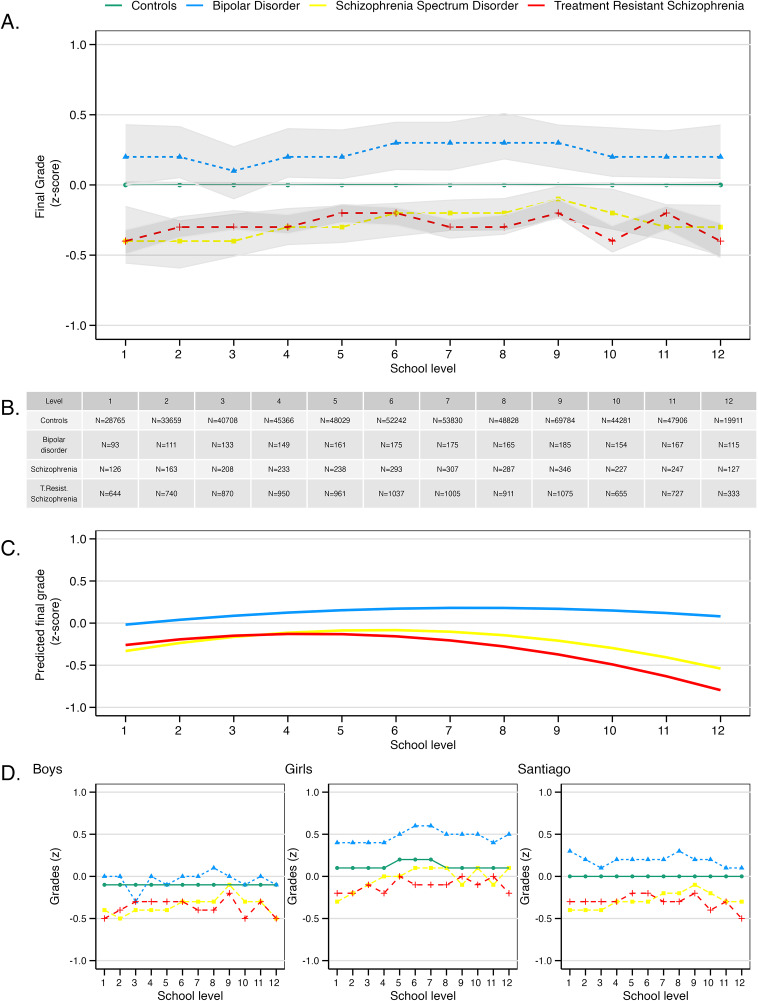
Grade trajectories according to school level, for children who were later diagnosed with treatment-resistant schizophrenia, treatment-responsive schizophrenia, bipolar disorder, and healthy controls. (a) Normalized (z-scored) grades for different school levels are presented with 95% confidence intervals, considering school and year attended, but not corrected for sex. (b) Number of participants per level. (c) Modeled trajectories for each group. (d) Normalized grades for boys, girls, and participants residing in Santiago.

Significant disparities in grades were observed among the groups studied. Figure 1a shows the normalized grades (z-scores) by school level, unadjusted for other confounders. Since groups differed according to sex, we also present the data separated by sex in 1D. The model that best fit the data compared the different groups with their unaffected classmates, including the interaction term *group×age* and *group×age* ^2^ (Table S1).

Children later diagnosed with treatment-resistant schizophrenia consistently underperformed relative to their unaffected classmates, scoring −0.25 standard deviations (SD) lower than their peers (95% confidence interval (CI) −0.20 to −0.30, *P* <0.0001). Their trajectory showed a linear increase in grades with age (*group×age* = 0.07, 95% CI 0.06 to 0.09, *P* <0.0001), which was offset by a non-linear decrease with age (*group×age* ^2^ =−0.01, 95% CI −0.012 to −0.009, *P* <0.0001). As Figure 1b shows, this resulted in children developing treatment-resistant schizophrenia maintaining a relatively stable level of underperformance at a young age until around 13–14 years old, when their grades deteriorated further.

A similar picture was seen in children later diagnosed with schizophrenia who did not require clozapine. Compared to their healthy classmates, they scored −0.37 standard deviations (95% CI −0.26 to −0.47, *P* <0.0001) lower, with a trajectory characterized by a linear increase with age (*group×age* = 0.10, 95% CI 0.07 to 0.13, *P*<0.0001) offset by a non-linear decrease (*group*×*age* ^2^ =−0.01, 95% CI −0.013 to −0.008, *P* <0.0001) (Figure 1b). When compared to children who later developed treatment-resistant schizophrenia, the best-fitting model included only the linear interaction term between group and age (Table S2). The significant difference between these two groups was a faster linear decline in grades with age in those who developed treatment resistance (*group×age* = −0.03, 95% CI −0.04 to −0.01, *P* = 0.0008).

Children who developed bipolar disorder underperformed −0.17 SD (95% CI −0.31 to −0.02, *P* = 0.02) compared to their peers. However, this finding applied to boys only, as there was a significant interaction between bipolar disorder and female sex (*group×sex* = 0.31, 95% CI 0.16 to 0.46, *P*<0.0001), where girls outperformed their unaffected peers (Figure 1d). Trajectories followed a similar shape to the other two groups developing severe mental illness (Figure 1b), with a linear increase with age (*group×age* = 0.06, 96% CI 0.02 to 0.09, *P* =0.0006) and a non-linear decrease with age^2^ (*group×age* ^2^ = −0.003, 95% CI −0.006 to −0.0006, *P* =0.018). When compared to children who developed treatment-resistant schizophrenia, the best-fitting model included interaction terms between group and age, and group and age^2^ (Table S3). Girls who later developed treatment-resistant schizophrenia performed worse than girls who later developed bipolar disorder (*Group×Sex =* −0.32, 95% CI −0.50 to −0.14, *P* =0.0005), although this was not the case for boys. Children who later developed treatment resistance also had a larger decline in grades with age, with a significant interaction between group and age^2^ (*Group×Age* ^2^ = −0.005, 95% CI −0.01 to −0.001, *P* =0.01).

Full hierarchical models for the analyses described are reported in the Supplementary Information (Tables S4–S6). Results were substantially unchanged when restricting the analyses to individuals who developed treatment-resistant schizophrenia who had attended school in the main capital Santiago (Figure 1d and Supplementary Results).

### School attendance

**Figure 2.**
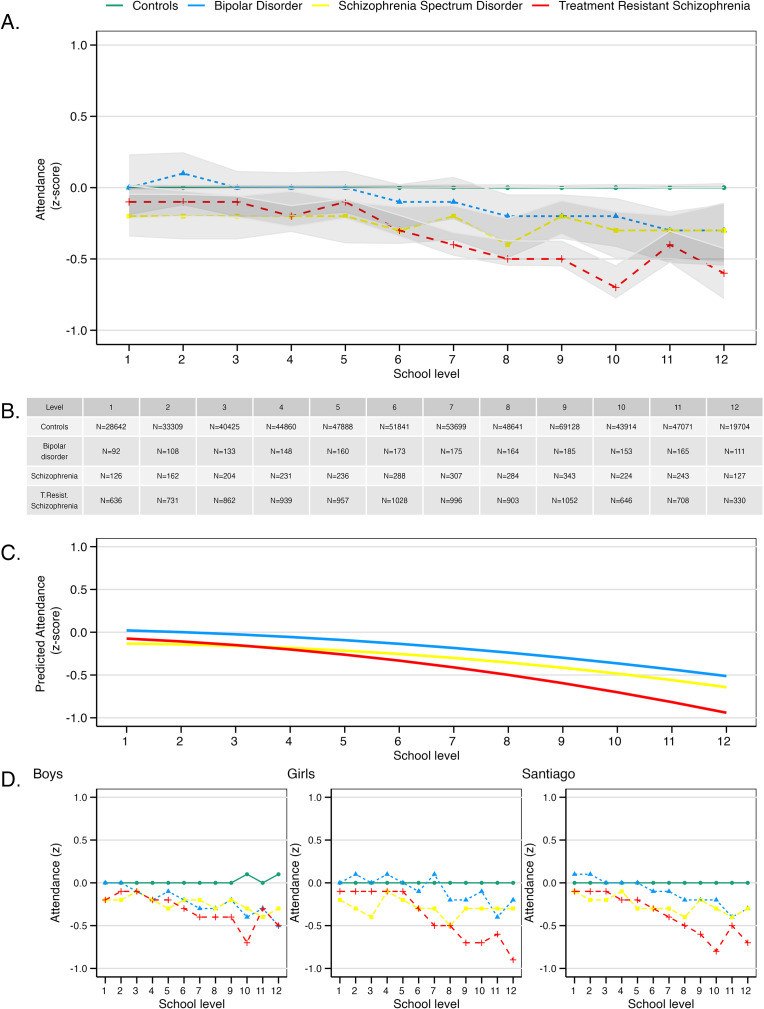
School attendance in children who were later diagnosed with treatment-resistant schizophrenia, treatment-responsive schizophrenia, bipolar disorder, and healthy controls, by school level. (a) Normalized (z-scored) attendance from 1st to 12th grade with 95% confidence intervals, considering school and year attended, but not corrected for sex. (b) Number of participants included per level. (c) Modeled trajectories for each group. (d) Normalized attendance for boys, girls, and participants residing in Santiago.

School attendance rates showed a decline over time among the three groups of children who were later diagnosed with severe mental illness (Figure 2a). The model that best fit these data included the interaction term *group×age* and *group×age* ^2^ (Table S7).

**Figure 3.**
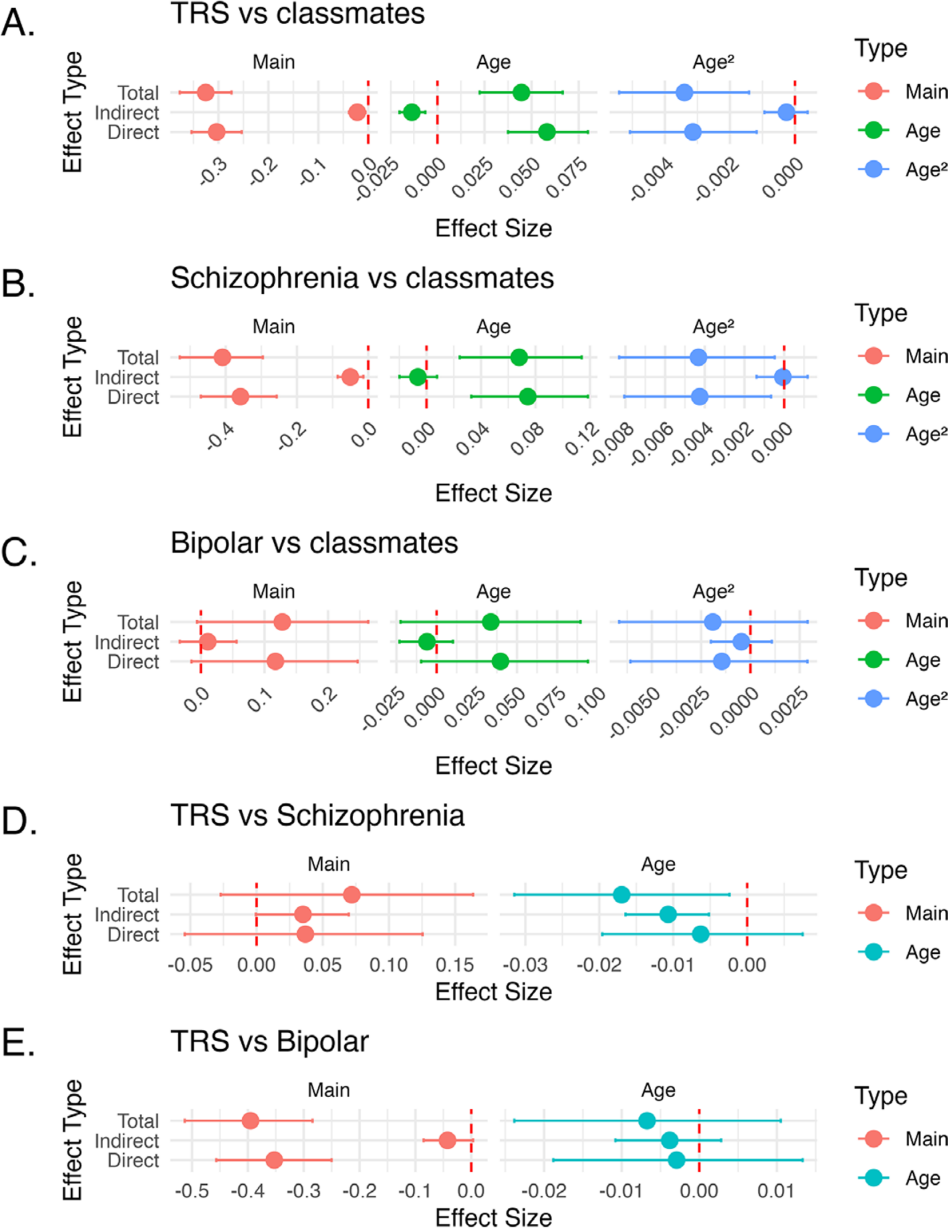
Mediation analyses showing total, indirect and direct effect of attendance on grades on the five comparisons (A-E). Note the small indirect effect of attendance across comparisons. The exception is the steeper decline in grades observed in treatment resistant groups compared to treatment-responsive schizophrenia, which is significantly driven by a steeper increase in non-attendance (D). TRS = treatment-resistant schizophrenia.

Girls who later developed treatment-resistant schizophrenia had significantly lower attendance rates than their unaffected peers (*group×sex* = −0.07, 95% CI −0.13 to −0.001, *P* = 0.04, Figure 2b). Boys showed a similar decrease in magnitude but it was not statistically significant. There was a marked difference in trajectories, with children who later developed treatment resistant schizophrenia decreasing in their attendance both linearly (*group×age* = −0.03, 95% CI −0.05 to −0.01, *P* = 0.009) and non-linearly (*group×age* ^2^ = −0.005, 95% CI −0.007 to −0.003, *P* <0.0001) compared to their peers (Figure 3b).

Children who developed treatment-responsive schizophrenia showed a significant non-linear decline in attendance compared to unaffected peers (*group×age* ^2^ = −0.004, 95%CI −0.007 to −0.0003, *P* = 0.035). The model that best fit the comparison between future treatment-resistant and treatment-responsive groups included the linear interaction of age and group (Table S8), with those developing treatment-resistant schizophrenia experiencing significantly faster decline in attendance with age (*group×age* = −0.03, 95% CI −0.05 to −0.01, *P* = 0.001).

No significant differences in attendance rates were observed between children who later developed bipolar disorder and their unaffected peers. When comparing them with children who developed treatment resistance, the best-fitting model included the linear interaction *group×age* (Table S9). Children who later developed treatment-resistant schizophrenia had a significantly faster decline in attendance with age compared to those developing bipolar disorder (*group×age* = −0.027, 95%CI −0.049 to −0.006, *P* = 0.012).

Full hierarchical models are reported in the Supplementary Information (Tables S10–S12), and sensitivity analyses including only individuals developing TRS from Santiago showing similar results are reported in the Supplementary Results.

### Mediation analyses

We examined whether non-attendance mediated the relationship between future development of severe mental illness and school grades. As Figure 3a–c shows, increased non-attendance explained a small part of the association between grades and the three severe mental disorders compared with their classmates, both for the main effects and also for the interaction effects with age (*age* and *age* ^2^). In other words, changes in attendance had little impact on the differences in grades seen between the three groups of children developing severe mental illness and their classmates.

Indirect effects of attendance on grades had a larger effect on grades when comparing TRS with the group developing treatment-responsive schizophrenia (Figure 3d) or bipolar disorder (Figure 3e). This was significant for the comparison TRS-schizophrenia, where the faster decline in grades in TRS was explained by a statistically significant indirect effect of reduced attendance. In other words, the faster decline of grades in TRS compared to treatment-responsive schizophrenia was driven by decreases in attendance rates.

## Discussion

Drawing on administrative data from Chile, we found that children who were subsequently diagnosed with schizophrenia requiring clozapine treatment consistently had worse grades than their healthy classmates, but not when compared to those later diagnosed with treatment-responsive schizophrenia. Girls who later developed treatment resistance performed worse than those who later developed bipolar disorder. In terms of academic trajectories, the three groups with severe mental illness showed a decrease in grades in the later school levels compared to their unaffected peers, with those developing treatment-resistant schizophrenia presenting a more pronounced decline than those later presenting treatment-responsive schizophrenia or bipolar disorder. In terms of school attendance, lower attendance was significant only for girls who later developed treatment-resistant schizophrenia compared to their unaffected peers. However, the two groups of children who subsequently developed schizophrenia showed a decline in attendance with time, with those developing treatment resistance showing the sharpest decline.

Our hypothesis that treatment-resistant schizophrenia is characterized by a more pronounced premorbid developmental impairment compared to treatment-responsive schizophrenia was not fully supported by our data. Both groups showed similarly lower grades than their peers in their early school levels, with differences emerging over time, and mediated by higher non-attendance rates. Our study benefited from measurements across different developmental stages, allowing us to reconstruct trajectories that indicated a consistent decline around adolescence for the three groups of children developing severe mental illness compared to their unaffected peers. This decline in school performance around adolescence in psychotic disorders has also been reported in other studies (Jonas et al., 2022; Mollon et al., 2018).

This deterioration was more pronounced in those developing treatment-resistant schizophrenia compared to those developing treatment-responsive schizophrenia or bipolar disorder. This process was mediated in part by increased non-attendance rates, possibly suggesting an effect of prodromal symptoms or the earlier presentation of the illness itself. These dynamic changes should also be assessed considering the data showing small differences in early adolescence for individuals who later present treatment-resistant schizophrenia, which become more pronounced in young adulthood (Kowalec et al., 2021). Such a divergent trajectory, already evident in the premorbid stages in treatment-resistant schizophrenia, is also consistent with the observed worse cognitive functioning at psychosis onset (Millgate et al., 2023), which is further exacerbated in chronic cases (Millgate et al., 2022). Our results suggest that treatment resistance may stem from an aggressive, deteriorating pathological process around adolescence, or otherwise by an abnormality in a late maturational process, rather than from a marked premorbid early developmental impairment. The biological mechanisms underlying this deterioration remain to be identified, including determining whether they are shared with other psychotic disorders or have distinct neurobiological correlates (Potkin et al., 2020).

We also found a decline in attendance rates during the later school levels among children developing schizophrenia, with a significantly larger decline in those developing treatment-resistant schizophrenia compared to those who did not require clozapine. A similar, albeit non-significant, decline was seen in those developing bipolar disorders. These decreasing attendance rates likely signal the first appearance of the illness or its prodrome, and mediate the decline in grades at least in those developing treatment-resistant schizophrenia. Due to the nature of the data and the anonymization process, we were not able to control for periods of disease onset with or without treatment. Our data cannot clarify whether grades decreased due to class absences or if a common mechanism in psychosis caused declining grades and increased non-attendance. A similar pattern was observed in the greater number of different schools attended by those developing severe mental illness. Regardless of the underlying mechanisms, rising non-attendance rates could potentially be used to identify populations at higher risk for developing a severe mental illness, consistent with previous studies linking school absenteeism to mental health issues (John et al., 2022).

Unlike children who later developed schizophrenia, girls who developed bipolar disorder had higher grades compared to their classmates. This better premorbid functioning aligns with findings from other studies that associate it with future bipolar disorder (Koenen et al., 2009). The diagnosis of bipolar disorder used here does not require the presence of psychotic features and does not differentiate those presenting only with hypomania (bipolar type II). Therefore, our findings are likely to represent a heterogeneous group of patients with bipolar disorder.

It is important to note that the group of children who were later prescribed clozapine was drawn from records covering 80% of the population in Chile. This resembles a population-wide study and provides valuable insights from individuals in the Global South, where such data are scarce. However, we did not conduct formal analyses to assess the extent or patterns of missing data or loss to follow-up. As such, the potential impact of missingness on our findings remains unquantified. Follow-up using the register was continued until participants had a median age of 24–27 years across groups. This implied that people who develop severe mental illness at a later age are under-represented in our study, alongside those who develop treatment resistance after several years of illness. We normalized the data using the classmates of each participant and adjusted the analysis, allowing us to control for factors such as socioeconomic status, school quality, and urban/rural setting. However, groups still differed in terms of sex and geographic distribution. To account for this, we included sex in our models, allowing for a variable effect across groups through an interaction term, and conducted subgroup analyses restricted to participants from Santiago. We acknowledge that there is a recognized under-prescription of clozapine for patients with treatment-resistant, which would make the groups of treatment-resistant and treatment-responsive patients more similar. However, our treatment-responsive group was restricted to a population treated at the Instituto Psiquiátrico Horwitz, where we have shown that delays in clozapine initiation and prescription are small (Mena et al., 2018).

Finally, we should note that grade point average is a metric affected by general cognitive function. Some studies suggest that TRS patients differ from treatment-responsive patients in specific cognitive functions rather than in general cognition (Kravariti et al., 2019; Millgate et al., 2022). However, the instrument available to measure scholastic achievement in this study does not allow to analyze whether there are differences in specific cognitive functions.

Overall, our results show that grades in children who later develop treatment-resistant schizophrenia reflect a similar level of impairment at an early age to those developing treatment-responsive schizophrenia. This is followed by a larger decline in later school levels, which is associated with deteriorating attendance rates. This suggests that treatment resistance may result from a more aggressive pathological process or a late maturation abnormality rather than a more pronounced early premorbid impairment, highlighting a potential critical target for intervention.

## Supporting information

Conejeros-Pavez et al. supplementary material Conejeros-Pavez et al. supplementary material

## Data Availability

The data supporting this study can be downloaded at the school level from the following link: School Data Download. To access the database containing individual test scores, student, parent, and teacher questionnaires, and mental health data, please visit the transparency platform: Transparency Platform.

## Supplementary material

The supplementary material for this article can be found at http://doi.org/10.1017/S0033291725101062.

## Analytic code availability

The code for preparing and analyzing data is available upon request from the corresponding authors.
