# Supplementary material for: Premorbid school performance trajectories in patients with treatment-resistant schizophrenia prescribed clozapine in the public health system in Chile: a case-control study, 2007–2020
Source: Psychol Med. 2025 Jul 21;55:e205. doi: 10.1017/S0033291725101062 (PMC12315655; doi:10.1017/S0033291725101062)
Supplement: Conejeros-Pavez et al. supplementary material [file S0033291725101062sup001.docx]

**Supplementary Material**

**Premorbid school performance trajectories in patients with treatment-resistant schizophrenia prescribed clozapine in the public health system in Chile: a case-control study, 2007-2020**

J Conejeros-Pavez, J Vasquez, C Diaz, C Mena, J Undurraga, A Gonzalez-Valderrama, S Claro, EA Undurraga^[[1]](#footnote-1)^, NA Crossley^*^

Contents

[Supplementary Methods 2](#_Toc194308177)

[Mediation analyses 2](#_Toc194308178)

[Supplementary Results 3](#_Toc194308179)

[Analyses including individuals from Santiago only 3](#_Toc194308180)

[Supplementary Figures 4](#_Toc194308181)

[Figure S1. Data anonymization process 4](#_Toc194308182)

[Supplementary Tables 5](#_Toc194308183)

[Table S1. Comparative performance metrics for mixed models examining grades across groups 5](#_Toc194308184)

[Table S2. Comparative performance metrics for mixed models examining grades between children who later developed treatment-resistant schizophrenia and those that developed treatment-responsive schizophrenia 5](#_Toc194308185)

[Table S3. Comparative performance metrics for mixed models examining grades between children who later developed treatment-resistant schizophrenia and those that developed bipolar disorder 5](#_Toc194308186)

[Table S4. Hierarchical models for grades across disorders 6](#_Toc194308187)

[Table S5. Hierarchical models comparing grades in children later developing treatment-resistant schizophrenia and treatment-responsive schizophrenia 7](#_Toc194308188)

[Table S6. Hierarchical models comparing grades in children later developing treatment resistant schizophrenia and bipolar disorder. 8](#_Toc194308189)

[Table S7. Comparative performance metrics for mixed models examining attendance across groups 9](#_Toc194308190)

[Table S8. Comparative performance metrics for mixed models examining attendance between children who later developed treatment-resistant schizophrenia and those that developed treatment-responsive schizophrenia 9](#_Toc194308191)

[Table S9. Comparative performance metrics for mixed models examining attendance between children who later developed treatment-resistant schizophrenia and those that developed bipolar disorder 9](#_Toc194308192)

[Table S10. Hierarchical models examining attendance across groups 10](#_Toc194308193)

[Table S11. Hierarchical models comparing attendance in children later developing treatment-resistant schizophrenia and treatment-responsive schizophrenia 11](#_Toc194308194)

[Table S12. Hierarchical models comparing attendance in children later developing treatment-resistant schizophrenia and bipolar disorder 12](#_Toc194308195)

[STROBE Statement—checklist of items that should be included in reports of observational studies 13](#_Toc194308196)

# Supplementary Methods

## Mediation analyses

To explore the role of non-attendance on changes in grade point averages, we performed analyses using Bayesian regression modelling on pairs of groups. Specifically, we examined the mediation of attendance on differences in grades in the following group comparisons 1) the group developing treatment resistance compared to their classmates, 2) the group developing schizophrenia compared to their classmates, and 3) the group developing bipolar disorder compared to the their classmates; as well as 4) the group developing treatment resistant compared to treatment-responsive schizophrenia, and 5) the group developing treatment resistant compared to those developing bipolar disorder.

For those five comparisons, we specified the following mediator model predicting the attendance rates (z-scores) as a function of *group* and the interactions *group x age* and *group x age*^2^:

[1]

$${attendance}_{i}=\alpha+\delta_{1}{group}_{i}+\delta_{2}{sex}_{i}+\delta_{3}{age}_{i}+\delta_{4}{age}_{i}^{2}+\delta_{5}{year retention}_{i}$$

$+\delta_{6}{(group x age)}_{i}{+\delta}_{7}{(group x {age}^{2})}_{i}+\varepsilon$

And the following outcome model predicting z-score grades as a function of group, attendance, and their interactions:

[2]

$${grades}_{i}=\alpha+{attendance}_{i}+\gamma_{1}{group}_{i}+\gamma_{2}{sex}_{i}+\gamma_{3}{age}_{i}+\gamma_{4}{age}_{i}^{2}+\gamma_{5}{year retention}_{i}$$

$+\gamma_{6}{(group x age)}_{i}{+\gamma}_{7}{(group x {age}^{2})}_{i}+\gamma_{8}{(attendance x age)}_{i}{+\gamma}_{9}{(attendance x {age}^{2})}_{i}+\eta$

where *ϵ* and *η* are residual errors. Models were fitted using **two Markov Chain Monte Carlo chains**, each with **2,000 iterations (including 1,000 warm-up).** The mediation effects for the main effect were computed as:

- **Indirect Effect:***a* *×* *b*. where *a* is the effect of *group* on *attendance* and *b* is the effect of *attendance* on *grades*;
- **Direct Effect:** *c*′, the coefficient of *group* in the outcome model; and
- **Total Effect:** *c*′+ (*a* *×* *b*).

For age-moderated mediation, we additionally calculated similar indirect, direct and total effects, considering their effect on the interaction term *group x age* and *group x age*^2^. For the comparisons between treatment-resistant schizophrenia and treatment-responsive schizophrenia, and also between treatment-resistant schizophrenia and bipolar disorder, we restricted the modelling to the linear interaction between *age* and *group* as the interaction with the quadratic term did not improve the fit as described in the main analyses.

Analyses were conducted using **the *brms* R library (version 2.22.0)**.

# Supplementary Results

## Analyses including individuals from Santiago only

Individuals identified as developing schizophrenia and requiring clozapine (TRS) were drawn from a nation-wide database, pairing them with their unaffected school peers. However, the control group of individuals who developed schizophrenia or bipolar disorder not requiring clozapine were drawn from a database including patients from the metropolitan area of Santiago, the highly urbanized capital city of Chile. We therefore performed sensitity analyses only including participants with TRS from Santiago (607 patients, 5,005 observations, 32% female).

*TRS compared to schizophrenia (treatment-responsive)*

For grades, the model that best fit the data comparing children who later developed schizophrenia and TRS-Santiago included a linear interaction between *group×age*. Similar to the main results on the manuscript, children who developed TRS differed only in their trajectory to those who were treatment-responsive, with a steeper linear decline with age (*group×age* = -0.03, 95% CI -0.05 to -0.02).

For attendance, the model that best fit the data also included a linear interaction betwee *group×age*. Attendance in children who developed TRS from Santiago was 0.17SD (95% CI 0.01 to 0.32) higher than those who developed treatment-responsive schizophrenia, with a significant linear decline with age (*group×age* = -0.05, 95% CI -0.07 to -0.03).

*TRS compared to bipolar disorder*

For grades, the model that best fit the data comparing children who later developed bipolar disorder and TRS-Santiago included a linear interaction betwee *group×age* and a non-linear *group×age^2^.* Echoing the main results on the manuscript, Girls who later developed treatment-resistant schizophrenia performed worse than girls who later developed bipolar disorder (*Group×Sex =* -0.4, 95% CI -0.59 to -0.21), although this was not the case for boys. Children who later developed treatment-resistance also had a steeper decline in grades with age, with a significant interaction between group and age^2^ (*Group×Age^2^* = -0.006, 95% CI -0.01 to -0.001).

For attendance, the model that best fit the data also included a linear interaction betwee *group×age*. Attendance in girls who developed TRS from Santiago was lower than girls who developed bipolar disorder (*Group×Sex =* -0.22, 95% CI -0.42 to -0.02). There was also a significant linear decline with age in the whole group who developed TRS compared to bipolar disorder (*group×age* = -0.05, 95% CI -0.07 to -0.02).

# Supplementary Figures

## Figure S1. Data anonymization process

RUT is the national ID number in Chile, while M-RUN is the unique pseudo-anonymized identifier used in educational databases in Chile available for researchers. The process from RUT to M-RUN is only managed by the Ministry of Education.

# Supplementary Tables

## Table S1. Comparative performance metrics for mixed models examining grades across groups

| Model | Nr. of Parameters | AIC | BIC | Log- Likelihood | χ^2^ | Degrees of freedom | *P*-value |
| --- | --- | --- | --- | --- | --- | --- | --- |
| Basic model (BM) | 14 | 1275757 | 1275914 | -637864 |  |  |  |
| BM + *Group×Age* | 17 | 1275651 | 1275841 | -637808 | 111.98 | 3 | <0.0001 |
| **BM + *Group×Age^2^*** | **20** | **1275381** | **1275606** | **-637671** | **275.25** | **3** | **<0.0001** |

Pairwise comparisons using ANOVA between the Basic Model (BM) and the BM with linear interaction with age; and BM with linear interaction compared to BM with linear and quadratic interaction with age. Model selection with the best fit in bold.

## Table S2. Comparative performance metrics for mixed models examining grades between children who later developed treatment-resistant schizophrenia and those that developed treatment-responsive schizophrenia

| Model | Nr. of Parameters | AIC | BIC | Log- Likelihood | χ^2^ | Degrees of freedom | *P*-value |
| --- | --- | --- | --- | --- | --- | --- | --- |
| Basic model (BM) | 10 | 32740 | 32815 | -16360 |  |  |  |
| **BM + *Group×Age*** | **11** | **32731** | **32813** | **-16354** | **11.3067** | **1** | **0.0008** |
| BM + *Group×Age^2^* | 12 | 32733 | 32822 | -16354 | 0.0119 | 1 | 0.91 |

Pairwise comparisons using ANOVA between the Basic Model (BM) and the BM with linear interaction with age; and BM with linear interaction compared to BM with linear and quadratic interaction with age. Model selection with the best fit in bold.

## Table S3. Comparative performance metrics for mixed models examining grades between children who later developed treatment-resistant schizophrenia and those that developed bipolar disorder

| Model | Nr. of Parameters | AIC | BIC | Log- Likelihood | χ^2^ | Degrees of freedom | *P*-value |
| --- | --- | --- | --- | --- | --- | --- | --- |
| Basic model (BM) | 10 | 29876 | 29950 | -14928 |  |  |  |
| BM + *Group×Age* | 11 | 29849 | 29930 | -14914 | 28.429 | 1 | <0.0001 |
| **BM + *Group×Age^2^*** | **12** | **29845** | **29933** | **-14910** | **6.506** | **1** | **0.011** |

Pairwise comparisons using ANOVA between the Basic Model (BM) and the BM with linear interaction with age; and BM with linear interaction compared to BM with linear and quadratic interaction with age. Model selection with the best fit in bold.

## Table S4. Hierarchical models for grades across disorders

|  | **Model 1** | | | **Model 2** | | | **Model 3** | | | **Model 4** | | | **Model 5** | | | **Model 6** | | |
| --- | --- | --- | --- | --- | --- | --- | --- | --- | --- | --- | --- | --- | --- | --- | --- | --- | --- | --- |
|  | *Beta* | *SE* | *P-value* | *Beta* | *SE* | *P-value* | *Beta* | *SE* | *P-value* | *Beta* | *SE* | *P-value* | *Beta* | *SE* | *P-value* | *Beta* | *SE* | *P-value* |
| (Intercept) | **-0.1072** | **0.0036** | **<0.0001** | **-0.1330** | **0.0040** | **<0.0001** | **-0.1324** | **0.0040** | **<0.0001** | **-0.1321** | **0.0040** | **<0.0001** | **-0.1360** | **0.0040** | **<0.0001** | **-0.1304** | **0.0040** | **<0.0001** |
| Treatment resist. Schizophrenia | **-0.2626** | **0.0162** | **<0.0001** | **-0.2628** | **0.0162** | **<0.0001** | **-0.2652** | **0.0163** | **<0.0001** | **-0.2618** | **0.0191** | **<0.0001** | **-0.1119** | **0.0243** | **<0.0001** | **-0.2494** | **0.0261** | **<0.0001** |
| Schizophrenia | **-0.1900** | **0.0295** | **<0.0001** | **-0.1910** | **0.0295** | **<0.0001** | **-0.1931** | **0.0295** | **<0.0001** | **-0.2109** | **0.0337** | **<0.0001** | **-0.1876** | **0.0470** | **0.0001** | **-0.3667** | **0.0524** | **<0.0001** |
| Bipolar disorder | **0.1827** | **0.0399** | **<0.0001** | **0.1844** | **0.0399** | **<0.0001** | **0.1836** | **0.0399** | **<0.0001** | 0.0178 | 0.0578 | 0.7580 | -0.1058 | 0.0682 | 0.1210 | **-0.1687** | **0.0733** | **0.0214** |
| Sex (female) | **0.2500** | **0.0036** | **<0.0001** | **0.2519** | **0.0036** | **<0.0001** | **0.2521** | **0.0036** | **<0.0001** | **0.2514** | **0.0036** | **<0.0001** | **0.2512** | **0.0036** | **<0.0001** | **0.2509** | **0.0036** | **<0.0001** |
| Age | **-0.0091** | **0.0004** | **<0.0001** | **0.0081** | **0.0012** | **<0.0001** | **0.0078** | **0.0012** | **<0.0001** | **0.0078** | **0.0012** | **<0.0001** | **0.0086** | **0.0012** | **<0.0001** | **0.0050** | **0.0012** | **0.0001** |
| Age^2^ |  |  |  | **-0.0017** | **0.0001** | **<0.0001** | **-0.0017** | **0.0001** | **<0.0001** | **-0.0017** | **0.0001** | **<0.0001** | **-0.0017** | **0.0001** | **<0.0001** | **-0.0014** | **0.0001** | **<0.0001** |
| School year retention |  |  |  |  |  |  | **0.0248** | **0.0042** | **<0.0001** | **0.0248** | **0.0042** | **<0.0001** | **0.0241** | **0.0042** | **<0.0001** | **0.0242** | **0.0042** | **<0.0001** |
| TRS x Sex |  |  |  |  |  |  |  |  |  | -0.0124 | 0.0354 | 0.7255 | -0.0131 | 0.0354 | 0.7116 | -0.0109 | 0.0354 | 0.7584 |
| Schizophr x Sex |  |  |  |  |  |  |  |  |  | 0.0710 | 0.0658 | 0.2803 | 0.0708 | 0.0658 | 0.2818 | 0.0803 | 0.0658 | 0.2223 |
| Bipolar x Sex |  |  |  |  |  |  |  |  |  | **0.3053** | **0.0769** | **0.0001** | **0.3083** | **0.0769** | **0.0001** | **0.3117** | **0.0769** | **0.0001** |
| TRS x Age |  |  |  |  |  |  |  |  |  |  |  |  | **-0.0266** | **0.0027** | **<0.0001** | **0.0734** | **0.0073** | **<0.0001** |
| Schizophr x Age |  |  |  |  |  |  |  |  |  |  |  |  | -0.0037 | 0.0053 | 0.4765 | **0.1018** | **0.0146** | **<0.0001** |
| Bipolar x Age |  |  |  |  |  |  |  |  |  |  |  |  | **0.0201** | **0.0059** | **0.0007** | **0.0571** | **0.0167** | **0.0006** |
| TRS x Age^2^ |  |  |  |  |  |  |  |  |  |  |  |  |  |  |  | **-0.0102** | **0.0007** | **<0.0001** |
| Schizophr x Age^2^ |  |  |  |  |  |  |  |  |  |  |  |  |  |  |  | **-0.0101** | **0.0013** | **<0.0001** |
| Bipolar x Age^2^ |  |  |  |  |  |  |  |  |  |  |  |  |  |  |  | **-0.0035** | **0.0015** | **0.0176** |
| *Random effects* |  |  |  |  |  |  |  |  |  |  |  |  |  |  |  |  |  |  |
| Individual: School (std. dev.) |  | 0.8773 |  |  | 0.8779 |  |  | 0.8780 |  |  | 0.8780 |  |  | 0.8781 |  |  | 0.8783 |  |
| School (std. dev.) |  | 0.0267 |  |  | 0.0324 |  |  | 0.0332 |  |  | 0.0333 |  |  | 0.0326 |  |  | 0.0320 |  |
| Error |  | 0.4706 |  |  | 0.4701 |  |  | 0.4701 |  |  | 0.4701 |  |  | 0.4699 |  |  | 0.4696 |  |
| AIC |  | 1276031 |  |  | 1275800 |  |  | 1275768 |  |  | 1275757 |  |  | 1275651 |  |  | 1275381 |  |
| Observations |  | 547802 |  |  | 547802 |  |  | 547802 |  |  | 547802 |  |  | 547802 |  |  | 547802 |  |

TRS= Treatment Resistant Schizophrenia. Std.Dev= standard deviation. SE= standard error. AIC=Akaike information criterion.

## Table S5. Hierarchical models comparing grades in children later developing treatment-resistant schizophrenia and treatment-responsive schizophrenia

|  |  | **Model 1** | |  | **Model 2** | |  | **Model 3** | |  | **Model 4** | |  | **Model 5** | |  | **Model 6** | |
| --- | --- | --- | --- | --- | --- | --- | --- | --- | --- | --- | --- | --- | --- | --- | --- | --- | --- | --- |
|  | Beta | SE | P-value | Beta | SE | P-value | Beta | SE | P-value | Beta | SE | P-value | Beta | SE | P-value | Beta | SE | P-value |
| (Intercept) | **-0.2217** | **0.0376** | **<0.0001** | **-0.3648** | **0.0399** | **<0.0001** | **-0.3820** | **0.0400** | **<0.0001** | **-0.3995** | **0.0430** | **<0.0001** | **-0.5283** | **0.0576** | **<0.0001** | **-0.5314** | **0.0644** | **<0.0001** |
| Treatment resist. Schizophrenia | **-0.0765** | **0.0355** | **0.0311** | **-0.0758** | **0.0358** | **0.0345** | **-0.0783** | **0.0360** | **0.0295** | -0.0554 | 0.0415 | 0.1814 | 0.0994 | 0.0619 | 0.1087 | 0.1032 | 0.0711 | 0.1467 |
| Sex (female) | **0.2641** | **0.0330** | **<0.0001** | **0.2674** | **0.0333** | **<0.0001** | **0.2711** | **0.0334** | **<0.0001** | **0.3407** | **0.0709** | **<0.0001** | **0.3421** | **0.0710** | **<0.0001** | **0.3422** | **0.0710** | **<0.0001** |
| Age | **-0.0215** | **0.0031** | **<0.0001** | **0.0750** | **0.0088** | **<0.0001** | **0.0752** | **0.0088** | **<0.0001** | **0.0753** | **0.0088** | **<0.0001** | **0.0977** | **0.0110** | **<0.0001** | **0.0995** | **0.0197** | **<0.0001** |
| Age^2^ |  |  |  | **-0.0097** | **0.0008** | **<0.0001** | **-0.0097** | **0.0008** | **<0.0001** | **-0.0097** | **0.0008** | **<0.0001** | **-0.0099** | **0.0008** | **<0.0001** | **-0.0101** | **0.0017** | **<0.0001** |
| School year retention |  |  |  |  |  |  | **0.1534** | **0.0260** | **<0.0001** | **0.1532** | **0.0260** | **<0.0001** | **0.1540** | **0.0260** | **<0.0001** | **0.1540** | **0.0260** | **<0.0001** |
| TRS x Sex |  |  |  |  |  |  |  |  |  | -0.0892 | 0.0802 | 0.2665 | -0.0907 | 0.0803 | 0.2584 | -0.0909 | 0.0803 | 0.2576 |
| TRS x Age |  |  |  |  |  |  |  |  |  |  |  |  | **-0.0256** | **0.0076** | **0.0008** | -0.0278 | 0.0220 | 0.2059 |
| TRS x Age^2^ |  |  |  |  |  |  |  |  |  |  |  |  |  |  |  | 0.0002 | 0.0020 | 0.9131 |
| *Random effects* |  |  |  |  |  |  |  |  |  |  |  |  |  |  |  |  |  |  |
| Individual: School (std. dev.) |  | 0.8375 |  |  | 0.8470 |  |  | 0.8567 |  |  | 0.8563 |  |  | 0.8577 |  |  | 0.8576 |  |
| School (std. dev.) |  | 0.2185 |  |  | 0.2357 |  |  | 0.2232 |  |  | 0.2244 |  |  | 0.2215 |  |  | 0.2215 |  |
| Error |  | 0.6699 |  |  | 0.6613 |  |  | 0.6583 |  |  | 0.6583 |  |  | 0.6577 |  |  | 0.6577 |  |
| AIC |  | 32906 |  |  | 32772 |  |  | 32739 |  |  | 32740 |  |  | 32731 |  |  | 32733 |  |
| Observations |  | 12710 |  |  | 12710 |  |  | 12710 |  |  | 12710 |  |  | 12710 |  |  | 12710 |  |

TRS= Treatment Resistant Schizophrenia. Std.Dev= standard deviation. SE= standard error. AIC=Akaike information criterion. In bold factors significant at <0.05 uncorrected.

## Table S6. Hierarchical models comparing grades in children later developing treatment resistant schizophrenia and bipolar disorder.

|  |  | **Model 1** | |  | **Model 2** | |  | **Model 3** | |  | **Model 4** | |  | **Model 5** | |  | **Model 6** | |
| --- | --- | --- | --- | --- | --- | --- | --- | --- | --- | --- | --- | --- | --- | --- | --- | --- | --- | --- |
|  | Beta | SE | P-value | Beta | SE | P-value | Beta | SE | P-value | Beta | SE | P-value | Beta | SE | P-value | Beta | SE | P-value |
| (Intercept) | **0.1246** | **0.0501** | **0.0130** | 0.0061 | 0.0518 | 0.9055 | -0.0050 | 0.0520 | 0.9227 | **-0.1489** | **0.0665** | **0.0252** | **-0.3846** | **0.0799** | **<0.0001** | **-0.2997** | **0.0866** | **0.0005** |
| Treatment resist. Schizophrenia | **-0.4370** | **0.0467** | **<0.0001** | **-0.4459** | **0.0470** | **<0.0001** | **-0.4527** | **0.0473** | **<0.0001** | **-0.2940** | **0.0658** | **<0.0001** | -0.0226 | 0.0832 | 0.7863 | -0.1214 | 0.0919 | 0.1867 |
| Sex (female) | **0.2955** | **0.0345** | **<0.0001** | **0.2980** | **0.0348** | **<0.0001** | **0.3020** | **0.0349** | **<0.0001** | **0.5661** | **0.0839** | **<0.0001** | **0.5720** | **0.0842** | **<0.0001** | **0.5674** | **0.0843** | **<0.0001** |
| Age | **-0.0203** | **0.0031** | **<0.0001** | **0.0658** | **0.0089** | **<0.0001** | **0.0659** | **0.0089** | **<0.0001** | **0.0661** | **0.0089** | **<0.0001** | **0.1077** | **0.0118** | **<0.0001** | **0.0591** | **0.0224** | **0.0084** |
| Age^2^ |  |  |  | **-0.0086** | **0.0008** | **<0.0001** | **-0.0086** | **0.0008** | **<0.0001** | **-0.0086** | **0.0008** | **<0.0001** | **-0.0091** | **0.0008** | **<0.0001** | **-0.0046** | **0.0019** | **0.0188** |
| School year retention |  |  |  |  |  |  | **0.1366** | **0.0275** | **<0.0001** | **0.1363** | **0.0275** | **<0.0001** | **0.1376** | **0.0274** | **<0.0001** | **0.1386** | **0.0274** | **<0.0001** |
| TRS x Sex |  |  |  |  |  |  |  |  |  | **-0.3189** | **0.0922** | **0.0005** | **-0.3249** | **0.0925** | **0.0004** | **-0.3202** | **0.0925** | **0.0005** |
| TRS x Age |  |  |  |  |  |  |  |  |  |  |  |  | **-0.0450** | **0.0084** | **<0.0001** | 0.0135 | 0.0244 | 0.5812 |
| TRS x Age^2^ |  |  |  |  |  |  |  |  |  |  |  |  |  |  |  | **-0.0055** | **0.0021** | **0.0107** |
| *Random effects* |  |  |  |  |  |  |  |  |  |  |  |  |  |  |  |  |  |  |
| Individual: School (std. dev.) |  | 0.8820 |  |  | 0.8888 |  |  | 0.8971 |  |  | 0.8952 |  |  | 0.8992 |  |  | 0.9005 |  |
| School (std. dev.) |  | 0.1126 |  |  | 0.1385 |  |  | 0.1198 |  |  | 0.1209 |  |  | 0.1210 |  |  | 0.1200 |  |
| Error |  | 0.6575 |  |  | 0.6507 |  |  | 0.6483 |  |  | 0.6483 |  |  | 0.6462 |  |  | 0.6456 |  |
| AIC |  | 30012 |  |  | 29908 |  |  | 29886 |  |  | 29876 |  |  | 29849 |  |  | 29845 |  |
| Observations |  | 11691 |  |  | 11691 |  |  | 11691 |  |  | 11691 |  |  | 11691 |  |  | 11691 |  |

TRS= Treatment Resistant Schizophrenia. Std.Dev= standard deviation. SE= standard error. AIC=Akaike information criterion. In bold factors significant at <0.05 uncorrected.

## Table S7. Comparative performance metrics for mixed models examining attendance across groups

| Model | Nr. of Parameters | AIC | BIC | Log- Likelihood | χ^2^ | Degrees of freedom | *P*-value |
| --- | --- | --- | --- | --- | --- | --- | --- |
| Basic model (BM) | 14 | 1445860 | 1446017 | -722916 |  |  |  |
| BM + *Group×Age* | 17 | 1445327 | 1445518 | -722647 | 538.70 | 3 | <0.0001 |
| **BM + *Group×Age^2^*** | **20** | **1445303** | **1445528** | **-722632** | **29.695** | **3** | **<0.0001** |

Pairwise comparisons using ANOVA between the Basic Model ad BM with linear interaction with age; and BM with linear interaction compared to BM with linear and quadratic interaction with age. Model selection with the best fit in bold.

## Table S8. Comparative performance metrics for mixed models examining attendance between children who later developed treatment-resistant schizophrenia and those that developed treatment-responsive schizophrenia

| Model | Nr. of Parameters | AIC | BIC | Log- Likelihood | χ^2^ | Degrees of freedom | *P*-value |
| --- | --- | --- | --- | --- | --- | --- | --- |
| Basic model (BM) | 10 | 38919 | 38994 | -19450 |  |  |  |
| **BM + *Group×Age*** | **11** | **38911** | **38993** | **-19444** | **10.4315** | **1** | **0.0012** |
| BM + *Group×Age^2^* | 12 | 38913 | 39002 | -19444 | 0.0455 | 1 | 0.83 |

Pairwise comparisons using ANOVA between the Basic Model ad BM with linear interaction with age; and BM with linear interaction compared to BM with linear and quadratic interaction with age. Model selection with the best fit in bold.

## Table S9. Comparative performance metrics for mixed models examining attendance between children who later developed treatment-resistant schizophrenia and those that developed bipolar disorder

| Model | Nr. of Parameters | AIC | BIC | Log- Likelihood | χ^2^ | Degrees of freedom | *P*-value |
| --- | --- | --- | --- | --- | --- | --- | --- |
| Basic model (BM) | 35604 | 35678 | -17792 | 35584 |  |  |  |
| **BM + *Group×Age*** | **35600** | **35681** | **-17789** | **35578** | **6.366** | **1** | **0.012** |
| BM + *Group×Age^2^* | 35602 | 35690 | -17789 | 35578 | 0.1289 | 1 | 0.71 |

Pairwise comparisons using ANOVA between the Basic Model ad BM with linear interaction with age; and BM with linear interaction compared to BM with linear and quadratic interaction with age. Model selection with the best fit in bold.

## Table S10. Hierarchical models examining attendance across groups

|  |  | **Model 1** | |  | **Model 2** | |  | **Model 3** | |  | **Model 4** | |  | **Model 5** | |  | **Model 6** | |
| --- | --- | --- | --- | --- | --- | --- | --- | --- | --- | --- | --- | --- | --- | --- | --- | --- | --- | --- |
|  | Beta | SE | P-value | Beta | SE | P-value | Beta | SE | P-value | Beta | SE | P-value | Beta | SE | P-value | Beta | SE | P-value |
| (Intercept) | **-0.0103** | **0.0037** | **0.0051** | **-0.0096** | **0.0044** | **0.0301** | **-0.0097** | **0.0044** | **0.0278** | **-0.0100** | **0.0044** | **0.0237** | **-0.0219** | **0.0045** | **<0.0001** | **-0.0195** | **0.0045** | **<0.0001** |
| Treatment resist. Schizophrenia | **-0.3831** | **0.0150** | **<0.0001** | **-0.3831** | **0.0150** | **<0.0001** | **-0.3823** | **0.0150** | **<0.0001** | **-0.3633** | **0.0178** | **<0.0001** | 0.0468 | 0.0260 | 0.0723 | -0.0241 | 0.0299 | 0.4188 |
| Schizophrenia | **-0.3104** | **0.0273** | **<0.0001** | **-0.3104** | **0.0273** | **<0.0001** | **-0.3097** | **0.0273** | **<0.0001** | **-0.3041** | **0.0315** | **<0.0001** | -0.0313 | 0.0518 | 0.5463 | -0.1042 | 0.0624 | 0.0947 |
| Bipolar disorder | **-0.1872** | **0.0363** | **<0.0001** | **-0.1873** | **0.0363** | **<0.0001** | **-0.1870** | **0.0363** | **<0.0001** | **-0.2331** | **0.0533** | **<0.0001** | 0.0560 | 0.0713 | 0.4323 | -0.0022 | 0.0820 | 0.9782 |
| Sex (female) | 0.0045 | 0.0034 | 0.1825 | 0.0045 | 0.0034 | 0.1831 | 0.0046 | 0.0034 | 0.1809 | 0.0051 | 0.0034 | 0.1338 | 0.0052 | 0.0034 | 0.1285 | 0.0052 | 0.0034 | 0.1300 |
| Age | **-0.0028** | **0.0005** | **<0.0001** | **-0.0032** | **0.0015** | **0.0360** | **-0.0031** | **0.0015** | **0.0443** | **-0.0031** | **0.0015** | **0.0445** | -0.0007 | 0.0016 | 0.6475 | -0.0022 | 0.0016 | 0.1698 |
| Age^2^ |  |  |  | 0.00003 | 0.0001 | 0.7837 | 0.00003 | 0.0001 | 0.8176 | 0.00003 | 0.0001 | 0.8183 | -0.00002 | 0.0001 | 0.8672 | 0.0001 | 0.0001 | 0.4223 |
| School year retention |  |  |  |  |  |  | -0.0092 | 0.0058 | 0.1115 | -0.0092 | 0.0058 | 0.1096 | -0.0105 | 0.0058 | 0.0682 | -0.0103 | 0.0058 | 0.0741 |
| TRS x Sex |  |  |  |  |  |  |  |  |  | **-0.0666** | **0.0330** | **0.0438** | **-0.0677** | **0.0330** | **0.0405** | **-0.0667** | **0.0330** | **0.0434** |
| Schizophr x Sex |  |  |  |  |  |  |  |  |  | -0.0216 | 0.0621 | 0.7277 | -0.0261 | 0.0621 | 0.6744 | -0.0233 | 0.0622 | 0.7076 |
| Bipolar x Sex |  |  |  |  |  |  |  |  |  | 0.0849 | 0.0720 | 0.2382 | 0.0774 | 0.0720 | 0.2821 | 0.0803 | 0.0720 | 0.2652 |
| TRS x Age |  |  |  |  |  |  |  |  |  |  |  |  | **-0.0737** | **0.0034** | **<0.0001** | **-0.0267** | **0.0103** | **0.0094** |
| Schizophr x Age |  |  |  |  |  |  |  |  |  |  |  |  | **-0.0444** | **0.0067** | **<0.0001** | -0.0038 | 0.0204 | 0.8534 |
| Bipolar x Age |  |  |  |  |  |  |  |  |  |  |  |  | **-0.0473** | **0.0077** | **<0.0001** | -0.0148 | 0.0238 | 0.5335 |
| TRS x Age^2^ |  |  |  |  |  |  |  |  |  |  |  |  |  |  |  | **-0.0047** | **0.0010** | **<0.0001** |
| Schizophr x Age^2^ |  |  |  |  |  |  |  |  |  |  |  |  |  |  |  | **-0.0038** | **0.0018** | **0.0350** |
| Bipolar x Age^2^ |  |  |  |  |  |  |  |  |  |  |  |  |  |  |  | -0.0030 | 0.0021 | 0.1493 |
| *Random effects* |  |  |  |  |  |  |  |  |  |  |  |  |  |  |  |  |  |  |
| Individual: School (std. dev.) |  | 0.7079 |  |  | 0.7079 |  |  | 0.7079 |  |  | 0.7079 |  |  | 0.7086 |  |  | 0.7088 |  |
| School (std. dev.) |  | 0 |  |  | 0 |  |  | 0 |  |  | 0 |  |  | 0 |  |  | 0 |  |
| Error |  | 0.7060 |  |  | 0.7060 |  |  | 0.7060 |  |  | 0.7060 |  |  | 0.7052 |  |  | 0.7051 |  |
| AIC |  | 1445858 |  |  | 1445860 |  |  | 1445859 |  |  | 1445860 |  |  | 1445327 |  |  | 1445303 |  |
| Observations |  | 543452 |  |  | 543452 |  |  | 543452 |  |  | 543452 |  |  | 543452 |  |  | 543452 |  |

TRS= Treatment Resistant Schizophrenia. Std.Dev= standard deviation. SE= standard error. AIC=Akaike information criterion. In bold factors significant at <0.05 uncorrected.

## Table S11. Hierarchical models comparing attendance in children later developing treatment-resistant schizophrenia and treatment-responsive schizophrenia

|  |  | **Model 1** | |  | **Model 2** | |  | **Model 3** | |  | **Model 4** | |  | **Model 5** | |  | **Model 6** | |
| --- | --- | --- | --- | --- | --- | --- | --- | --- | --- | --- | --- | --- | --- | --- | --- | --- | --- | --- |
|  | Beta | SE | P-value | Beta | SE | P-value | Beta | SE | P-value | Beta | SE | P-value | Beta | SE | P-value | Beta | SE | P-value |
| (Intercept) | 0.0768 | 0.0422 | 0.0690 | 0.0205 | 0.0460 | 0.6557 | 0.0285 | 0.0462 | 0.5372 | 0.0196 | 0.0492 | 0.6912 | -0.1368 | 0.0690 | 0.0474 | -0.1281 | 0.0802 | 0.1104 |
| Treatment resist. Schizophrenia | **-0.1045** | **0.0381** | **0.0061** | **-0.1042** | **0.0382** | **0.0064** | **-0.1033** | **0.0381** | **0.0068** | **-0.0917** | **0.0442** | **0.0380** | 0.0957 | 0.0729 | 0.1891 | 0.0850 | 0.0884 | 0.3363 |
| Sex (female) | -0.0534 | 0.0355 | 0.1322 | -0.0523 | 0.0356 | 0.1417 | -0.0544 | 0.0355 | 0.1260 | -0.0195 | 0.0758 | 0.7974 | -0.0166 | 0.0757 | 0.8262 | -0.0170 | 0.0757 | 0.8229 |
| Age | **-0.0648** | **0.0039** | **<0.0001** | **-0.0291** | **0.0120** | **0.0150** | **-0.0292** | **0.0120** | **0.0148** | **-0.0291** | **0.0120** | **0.0149** | **-0.0017** | **0.0147** | **0.9081** | **-0.0065** | **0.0268** | **0.8090** |
| Age^2^ |  |  |  | **-0.0035** | **0.0011** | **0.0015** | **-0.0035** | **0.0011** | **0.0016** | **-0.0035** | **0.0011** | **0.0016** | **-0.0037** | **0.0011** | **0.0007** | **-0.0033** | **0.0024** | **0.1638** |
| School year retention |  |  |  |  |  |  | **-0.0783** | **0.0353** | **0.0265** | **-0.0784** | **0.0353** | **0.0263** | **-0.0776** | **0.0353** | **0.0278** | **-0.0776** | **0.0353** | **0.0279** |
| TRS x Sex |  |  |  |  |  |  |  |  |  | -0.0447 | 0.0856 | 0.6018 | -0.0475 | 0.0855 | 0.5789 | -0.0471 | 0.0856 | 0.5817 |
| TRS x Age |  |  |  |  |  |  |  |  |  |  |  |  | **-0.0312** | **0.0097** | **0.0012** | -0.0251 | 0.0300 | 0.4015 |
| TRS x Age^2^ |  |  |  |  |  |  |  |  |  |  |  |  |  |  |  | -0.0006 | 0.0027 | 0.8310 |
| *Random effects* |  |  |  |  |  |  |  |  |  |  |  |  |  |  |  |  |  |  |
| Individual: School (std. dev.) |  | 0.7918 |  |  | 0.7942 |  |  | 0.7907 |  |  | 0.7904 |  |  | 0.7912 |  |  | 0.7913 |  |
| School (std. dev.) |  | 0.2116 |  |  | 0.2201 |  |  | 0.2263 |  |  | 0.2272 |  |  | 0.2212 |  |  | 0.2210 |  |
| Error |  | 0.9531 |  |  | 0.9513 |  |  | 0.9516 |  |  | 0.9516 |  |  | 0.9514 |  |  | 0.9513 |  |
| AIC |  | 38928 |  |  | 38920 |  |  | 38917 |  |  | 38919 |  |  | 38911 |  |  | 38913 |  |
| Observations |  | 12563 |  |  | 12563 |  |  | 12563 |  |  | 12563 |  |  | 12563 |  |  | 12563 |  |

TRS= Treatment Resistant Schizophrenia. Std.Dev= standard deviation. SE= standard error. AIC=Akaike information criterion. In bold factors significant at <0.05 uncorrected.

## Table S12. Hierarchical models comparing attendance in children later developing treatment-resistant schizophrenia and bipolar disorder

|  |  | **Model 1** | |  | **Model 2** | |  | **Model 3** | |  | **Model 4** | |  | **Model 5** | |  | **Model 6** | |
| --- | --- | --- | --- | --- | --- | --- | --- | --- | --- | --- | --- | --- | --- | --- | --- | --- | --- | --- |
|  | Beta | SE | P-value | Beta | SE | P-value | Beta | SE | P-value | Beta | SE | P-value | Beta | SE | P-value | Beta | SE | P-value |
| (Intercept) | **0.2161** | **0.0542** | **0.0001** | 0.1631 | 0.0571 | 0.0043 | 0.1673 | 0.0571 | 0.0034 | 0.1007 | 0.0717 | 0.1600 | -0.0437 | 0.0917 | 0.6339 | -0.0262 | 0.1039 | 0.8012 |
| Treatment resist. Schizophrenia | **-0.2347** | **0.0487** | **<0.0001** | **-0.2380** | **0.0489** | **<0.0001** | **-0.2352** | **0.0488** | **<0.0001** | **-0.1619** | **0.0683** | **0.0179** | 0.0030 | 0.0946 | 0.9749 | -0.0175 | 0.1104 | 0.8743 |
| Sex (female) | -0.0364 | 0.0365 | 0.3191 | -0.0355 | 0.0366 | 0.3332 | -0.0372 | 0.0366 | 0.3091 | 0.0849 | 0.0876 | 0.3327 | 0.0885 | 0.0877 | 0.3129 | 0.0877 | 0.0877 | 0.3176 |
| Age | **-0.0674** | **0.0040** | **<0.0001** | **-0.0316** | **0.0122** | **0.0096** | **-0.0316** | **0.0122** | **0.0096** | **-0.0315** | **0.0122** | **0.0099** | -0.0059 | 0.0158 | 0.7107 | -0.0156 | 0.0313 | 0.6189 |
| Age^2^ |  |  |  | **-0.0035** | **0.0011** | **0.0018** | **-0.0035** | **0.0011** | **0.0019** | **-0.0035** | **0.0011** | **0.0019** | **-0.0037** | **0.0011** | **0.0009** | -0.0029 | 0.0027 | 0.2893 |
| School year retention |  |  |  |  |  |  | -0.0608 | 0.0375 | 0.1050 | -0.0608 | 0.0375 | 0.1050 | -0.0600 | 0.0375 | 0.1093 | -0.0598 | 0.0375 | 0.1106 |
| TRS x Sex |  |  |  |  |  |  |  |  |  | -0.1476 | 0.0962 | 0.1252 | -0.1511 | 0.0963 | 0.1167 | -0.1502 | 0.0963 | 0.1189 |
| TRS x Age |  |  |  |  |  |  |  |  |  |  |  |  | **-0.0275** | **0.0109** | **0.0116** | -0.0159 | 0.0340 | 0.6389 |
| TRS x Age^2^ |  |  |  |  |  |  |  |  |  |  |  |  |  |  |  | -0.0011 | 0.0030 | 0.7195 |
| *Random effects* |  |  |  |  |  |  |  |  |  |  |  |  |  |  |  |  |  |  |
| Individual: School (std. dev.) |  | 0.7928 |  |  | 0.7954 |  |  | 0.7927 |  |  | 0.7920 |  |  | 0.7929 |  |  | 0.7931 |  |
| School (std. dev.) |  | 0.2305 |  |  | 0.2363 |  |  | 0.2401 |  |  | 0.2402 |  |  | 0.2408 |  |  | 0.2407 |  |
| Error |  | 0.9440 |  |  | 0.9423 |  |  | 0.9426 |  |  | 0.9427 |  |  | 0.9421 |  |  | 0.9420 |  |
| AIC |  | 35613 |  |  | 35605 |  |  | 35604 |  |  | 35604 |  |  | 35600 |  |  | 35602 |  |
| Observations |  | 11555 |  |  | 11555 |  |  | 11555 |  |  | 11555 |  |  | 11555 |  |  | 11555 |  |

TRS= Treatment Resistant Schizophrenia. Std.Dev= standard deviation. SE= standard error. AIC=Akaike information criterion. In bold factors significant at <0.05 uncorrected.

## STROBE Statement—checklist of items that should be included in reports of observational studies

|  | Item No. | | | Recommendation | Page  No. | | |
| --- | --- | --- | --- | --- | --- | --- | --- |
| **Title and abstract** | 1 | | | (*a*) Indicate the study’s design with a commonly used term in the title or the abstract | 1 | | |
|  |  |  |  | (*b*) Provide in the abstract an informative and balanced summary of what was done and what was found | 1 | | |
| Introduction | | | | | | | |
| Background/rationale | 2 | | | Explain the scientific background and rationale for the investigation being reported | 3-4 | | |
| Objectives | 3 | | | State specific objectives, including any prespecified hypotheses | 4 | | |
| Methods | | | | | | | |
| Study design | 4 | | | Present key elements of study design early in the paper | 5-6 | | |
| Setting | 5 | | | Describe the setting, locations, and relevant dates, including periods of recruitment, exposure, follow-up, and data collection | 5 | | |
| Participants | 6 | | | (*a*) *Cohort study*—Give the eligibility criteria, and the sources and methods of selection of participants. Describe methods of follow-up  *Case-control study*—Give the eligibility criteria, and the sources and methods of case ascertainment and control selection. Give the rationale for the choice of cases and controls  *Cross-sectional study*—Give the eligibility criteria, and the sources and methods of selection of participants | 5 | | |
|  |  |  |  | (*b*) *Cohort study*—For matched studies, give matching criteria and number of exposed and unexposed  *Case-control study*—For matched studies, give matching criteria and the number of controls per case | 5,8 | | |
| Variables | 7 | | | Clearly define all outcomes, exposures, predictors, potential confounders, and effect modifiers. Give diagnostic criteria, if applicable | 6 | | |
| Data sources/ measurement | 8* | | | For each variable of interest, give sources of data and details of methods of assessment (measurement). Describe comparability of assessment methods if there is more than one group | 6 | | |
| Bias | 9 | | | Describe any efforts to address potential sources of bias | 7 | | |
| Study size | 10 | | | Explain how the study size was arrived at | 5 | | |
| Quantitative variables | | 11 | Explain how quantitative variables were handled in the analyses. If applicable, describe which groupings were chosen and why | | 6 | | |
| Statistical methods | | 12 | (*a*) Describe all statistical methods, including those used to control for confounding | | 6 | | |
|  |  |  | (*b*) Describe any methods used to examine subgroups and interactions | | 6 | | |
|  |  |  | (*c*) Explain how missing data were addressed | | 6 | | |
|  |  |  | (*d*) *Cohort study*—If applicable, explain how loss to follow-up was addressed  *Case-control study*—If applicable, explain how matching of cases and controls was addressed  *Cross-sectional study*—If applicable, describe analytical methods taking account of sampling strategy | | 17 | | |
|  |  |  | (*e*) Describe any sensitivity analyses | | 7, SI | | |
| Results | | | | | | |  |
| Participants | | 13* | (a) Report numbers of individuals at each stage of study—eg numbers potentially eligible, examined for eligibility, confirmed eligible, included in the study, completing follow-up, and analysed | | 8 |  |  |
|  |  |  | (b) Give reasons for non-participation at each stage | | 17 |  |  |
|  |  |  | (c) Consider use of a flow diagram | |  |  |  |
| Descriptive data | | 14* | (a) Give characteristics of study participants (eg demographic, clinical, social) and information on exposures and potential confounders | | 8 |  |  |
|  |  |  | (b) Indicate number of participants with missing data for each variable of interest | | 17 |  |  |
|  |  |  | (c) *Cohort study*—Summarise follow-up time (eg, average and total amount) | |  |  |  |
| Outcome data | | 15* | *Cohort study*—Report numbers of outcome events or summary measures over time | |  |  |  |
|  |  |  | *Case-control study—*Report numbers in each exposure category, or summary measures of exposure | | 8 |  |  |
|  |  |  | *Cross-sectional study—*Report numbers of outcome events or summary measures | |  |  |  |
| Main results | | 16 | (*a*) Give unadjusted estimates and, if applicable, confounder-adjusted estimates and their precision (eg, 95% confidence interval). Make clear which confounders were adjusted for and why they were included | | 11,14 |  |  |
|  |  |  | (*b*) Report category boundaries when continuous variables were categorized | |  |  |  |
|  |  |  | (*c*) If relevant, consider translating estimates of relative risk into absolute risk for a meaningful time period | |  |  |  |
| Other analyses | | 17 | Report other analyses done—eg analyses of subgroups and interactions, and sensitivity analyses | | SI |  |  |
| Discussion | | | | | | |  |
| Key results | | 18 | Summarise key results with reference to study objectives | | 16 |  |  |
| Limitations | | 19 | Discuss limitations of the study, taking into account sources of potential bias or imprecision. Discuss both direction and magnitude of any potential bias | | 17 |  |  |
| Interpretation | | 20 | Give a cautious overall interpretation of results considering objectives, limitations, multiplicity of analyses, results from similar studies, and other relevant evidence | | 16-17 |  |  |
| Generalisability | | 21 | Discuss the generalisability (external validity) of the study results | | 17 |  |  |
| Other information | | |  | | | |  |
| Funding | | 22 | Give the source of funding and the role of the funders for the present study and, if applicable, for the original study on which the present article is based | | 18 |  |  |

*Give information separately for cases and controls in case-control studies and, if applicable, for exposed and unexposed groups in cohort and cross-sectional studies.

1. Corresponding authors: Eduardo A. Undurraga, Escuela de Gobierno, Pontificia Universidad Católica de Chile, Av. Vicuña Mackenna 4860, Macul, Santiago, RM 7820436, Chile; [eundurra@uc.cl](mailto:eundurra@uc.cl). Nicolas A. Crossley, Department of Psychiatry, School of Medicine, Pontificia Universidad Católica de Chile, Diagonal Paraguay 362, Santiago, RM 8330077, Chile; [ncrossley@uc.cl](mailto:ncrossley@uc.cl). [↑](#footnote-ref-1)
